# Copy number variants in fetuses with non-structural ultrasound abnormalities among pregnant women without adverse pregnancy history

**DOI:** 10.3389/fgene.2026.1908680

**Published:** 2026-09-28

**Authors:** Jianwen Lian, Liubing Lan, Wenjuan Luo, Lingna She, Bosen Zhang, Huaxian Wang, Hua Zhong, Zhiyuan Zheng, Heming Wu, Lifang Lin

**Affiliations:** 1 Department of Prenatal Diagnostic Center, Meizhou People’s Hospital, Meizhou Academy of Medical Sciences, Meizhou, China; 2 Meizhou Municipal Engineering and Technology Research Center for Molecular Diagnostics of Major Genetic Disorders, Meizhou People’s Hospital, Meizhou Academy of Medical Sciences, Meizhou, China; 3 Department of Obstetrics, Meizhou People’s Hospital, Meizhou Academy of Medical Sciences, Meizhou, China; 4 Center for Pathological Diagnostics, Meizhou People’s Hospital, Meizhou Academy of Medical Sciences, Meizhou, China; 5 Department of Ultrasound, Meizhou People’s Hospital, Meizhou Academy of Medical Sciences, Meizhou, China

**Keywords:** adverse pregnancy history, copy number variant, non-structural ultrasound abnormalities, prenatal diagnosis, ultrasound soft marker

## Abstract

**Objective:**

To analyze fetal copy number variants (CNVs) in pregnant women without adverse pregnancy history whose fetuses presented non-structural ultrasound abnormalities, and explore the correlations between CNVs status, ultrasonic phenotypes and maternal age.

**Methods:**

A total of 426 pregnant women without adverse pregnancy history were consecutively recruited prospectively, followed by retrospective data analysis. Participants were divided into <35 years (n = 368) and ≥35 years (n = 58) groups. All fetuses were classified by types of non-structural ultrasound abnormalities. Chromosomal microarray analysis (CMA) was used to detect pathogenic/likely pathogenic CNVs (P/LP CNVs) and variants of uncertain significance (VUS). Subgroup comparisons of CNV detection rates were performed.

**Results:**

Abnormal ultrasound soft markers were the most common manifestation (83.1%), followed by fetal growth restriction (12.2%) and abnormal amniotic fluid volume (4.2%). The overall detection rates of P/LP CNVs and VUS were 4.5% and 9.2%, respectively. The detection rates of P/LP CNVs and VUS in fetuses with abnormal ultrasound soft-markers were 3.4% and 9.6%, respectively. Both the detection rates of P/LP CNVs and VUS reached 9.6% in fetal growth restriction fetuses. The detection rate of P/LP CNVs was 5.6% in fetuses with abnormal amniotic fluid volume, whereas no VUS was detected. CNVs detection rates varied greatly among different soft markers: the detection rate was highest in fetuses with renal pelvic dilatation (33.3%, 5/15), followed by short long bones (17.9%, 5/28) and increased NT thickness (14.3%, 17/119). There were no significant differences in P/LP CNVs (*p* = 0.090) and VUS (*p* = 0.631) rates between pregnant women aged <35 years and ≥35 years.

**Conclusion:**

Fetal CNVs vary substantially across different non-structural ultrasound abnormalities in low-risk pregnancies. Fetal growth restriction and several ultrasound soft markers are associated with elevated CNVs risks. Maternal age has no significant impact on fetal CNVs detection. Stratified risk assessment and individualized management are recommended for this population.

## Introduction

Prenatal ultrasound is the cornerstone of routine fetal screening during pregnancy, which enables early identification of fetal developmental anomalies and provides critical evidence for prenatal genetic counseling and clinical decision-making (Sriraam et al., 2024). Non-structural fetal ultrasound abnormalities refer to imaging manifestations in prenatal ultrasonography without obvious malformations, defects or organic structural lesions in fetal anatomy, organs and body structure, yet with deviations from normal reference ranges in hemodynamics, rhythm, movement, appendages, physiological parameters and ultrasonic soft markers (Cai et al., 2020). They commonly include abnormal soft markers (Mangla and Kumar, 2025), fetal growth restriction (Schaak et al., 2024), and abnormal amniotic fluid volume (Hughes et al., 2020). Although these manifestations do not represent definite fetal organ malformations, they have been widely recognized as important risk indicators for fetal genetic disorders, attracting increasing attention from prenatal diagnosis specialists worldwide (Pan et al., 2023; Shi et al., 2022; Beke and Simonyi, 2025).

Copy number variants (CNVs) refer to gains (duplications) or losses (deletions) of DNA fragments ≥1 kb in the genome, which are submicroscopic genomic structural variants (Wafik et al., 2024). Pathogenic or likely pathogenic CNVs (P/LP CNVs) are closely associated with adverse fetal outcomes such as congenital malformations, intellectual disability, developmental delay and multiple system disorders (Kang et al., 2024). Variants of uncertain significance (VUS), another common type of CNV, cannot be clearly classified as benign or pathogenic at present, yet they also bring considerable confusion to prenatal counseling due to their potential clinical implications (Shi et al., 2023a). With the widespread application of chromosomal microarray analysis (CMA) in prenatal diagnosis, CMA is recommended as the first-tier genetic test for pregnancies with fetal ultrasound-detected structural abnormalities according to American College of Obstetricians and Gynecologists (ACOG), Society for Maternal-Fetal Medicine (SMFM), American College of Medical Genetics and Genomics (ACMG), and International Society of Ultrasound in Obstetrics and Gynecology (ISUOG) guidelines (Shao et al., 2021; Durham et al., 2019). Owing to its higher resolution relative to conventional karyotyping and capability to identify submicroscopic chromosomal imbalances including clinically relevant CNVs, CMA has substantially increased the diagnostic yield of fetal genetic diseases and reshaped standard prenatal diagnostic workflows (Krishnamurthy et al., 2025; Qin et al., 2025).

Most existing studies on the correlation between CNVs and fetal ultrasound abnormalities have primarily included fetuses from the general population receiving invasive prenatal diagnosis, namely, fetuses of pregnant women with advanced maternal age, and recurrent miscarriage, stillbirth, fetal malformations and other adverse pregnancy histories (Huang et al., 2025; Cai et al., 2026; Song et al., 2024). Such high-risk populations inherently have elevated baseline genetic risk, and population-level differences in baseline risk may confound reported associations between isolated non-structural ultrasound abnormalities and fetal CNVs. Notably, pregnant women without adverse pregnancy history are generally defined as low-risk pregnant populations (Wang Q. Q. et al., 2022). For this group, the clinical value and necessity of CNVs detection when fetal non-structural ultrasound abnormalities are detected remain controversial. Clarifying the distribution characteristics of CNVs in fetuses among pregnant women without adverse pregnancy history will help to optimize prenatal screening strategies and avoid excessive genetic testing for low-risk individuals.

Maternal age is also a well-established risk factor for fetal chromosomal and congenital anomalies (Pethő et al., 2024; Elmerdahl Frederiksen et al., 2024). Advanced maternal age (≥35 years old) is traditionally linked to an increased risk of fetal chromosomal abnormalities (Liu et al., 2025; La Marca et al., 2021). Nevertheless, it remains incompletely understood whether maternal age influences the detection rates of P/LP CNVs and VUS among fetuses with non-structural ultrasound abnormalities within low-risk pregnant populations without adverse pregnancy history. In addition, correlations between distinct ultrasound soft markers and pathogenic CNVs differ substantially, and CNV detection rates for individual soft markers still require validation from large-sample datasets (Pan et al., 2023; Hu et al., 2021). The aim is to analyze the CNVs in fetuses with non-structural ultrasound abnormalities in pregnant women without a history of adverse pregnancies, and to explore the correlations between CNV status, ultrasound phenotypes and the age of the pregnant women, in order to provide objective clinical data for genetic counseling and individualized prenatal diagnosis management in low-risk pregnancies complicated by fetal non-structural ultrasound abnormalities.

## Materials and methods

### Study cohort

This was a study with consecutive prospective recruitment of participants from October 2022 to December 2025, followed by retrospective data analysis. During the research period, we continuously recruited pregnant women with singleton pregnancies and fetuses showing non-structural ultrasound abnormalities, who had no adverse pregnancy history, and they all underwent prenatal genetic testing at Meizhou People’s Hospital. The study protocol was reviewed and approved by the Medical Ethics Committee of Meizhou People’s Hospital. Every participant was clearly informed of the objectives, testing procedures and clinical implications of prenatal genetic testing, and all provided signed written informed consent.

Inclusion criteria were as follows (Sriraam et al., 2024): pregnant women without adverse pregnancy history, including recurrent spontaneous abortion, stillbirth, neonatal death, fetal malformation, and history of children with genetic diseases (Cai et al., 2020); fetuses diagnosed with non-structural ultrasound abnormalities by prenatal ultrasonography (Mangla and Kumar, 2025); fetuses with normal karyotype (Schaak et al., 2024); complete baseline information (such as maternal age, gestational age at sampling, and history of adverse pregnancy), prenatal ultrasound reports and CNV detection results; and (Hughes et al., 2020) voluntarily accepted prenatal genetic testing and signed informed consent. Exclusion criteria (Sriraam et al., 2024): pregnant women with adverse pregnancy history (Cai et al., 2020); fetuses complicated with definite structural malformations (Mangla and Kumar, 2025); fetuses with abnormal chromosome karyotypes (Schaak et al., 2024); presence of maternal chromosomal abnormalities or known familial hereditary diseases; and (Hughes et al., 2020) incomplete clinical and laboratory data. A total of 426 fetal cases were ultimately included for data analysis. The definition of adverse pregnancy events covered prior spontaneous miscarriage, pregnancy termination caused by fetal defects, intrauterine fetal demise, neonatal mortality, and deliveries of children with congenital deformities or chromosomal abnormalities (Zhong et al., 2024). The participant screening and selection process is illustrated in the flowchart (Figure 1).

**FIGURE 1:**
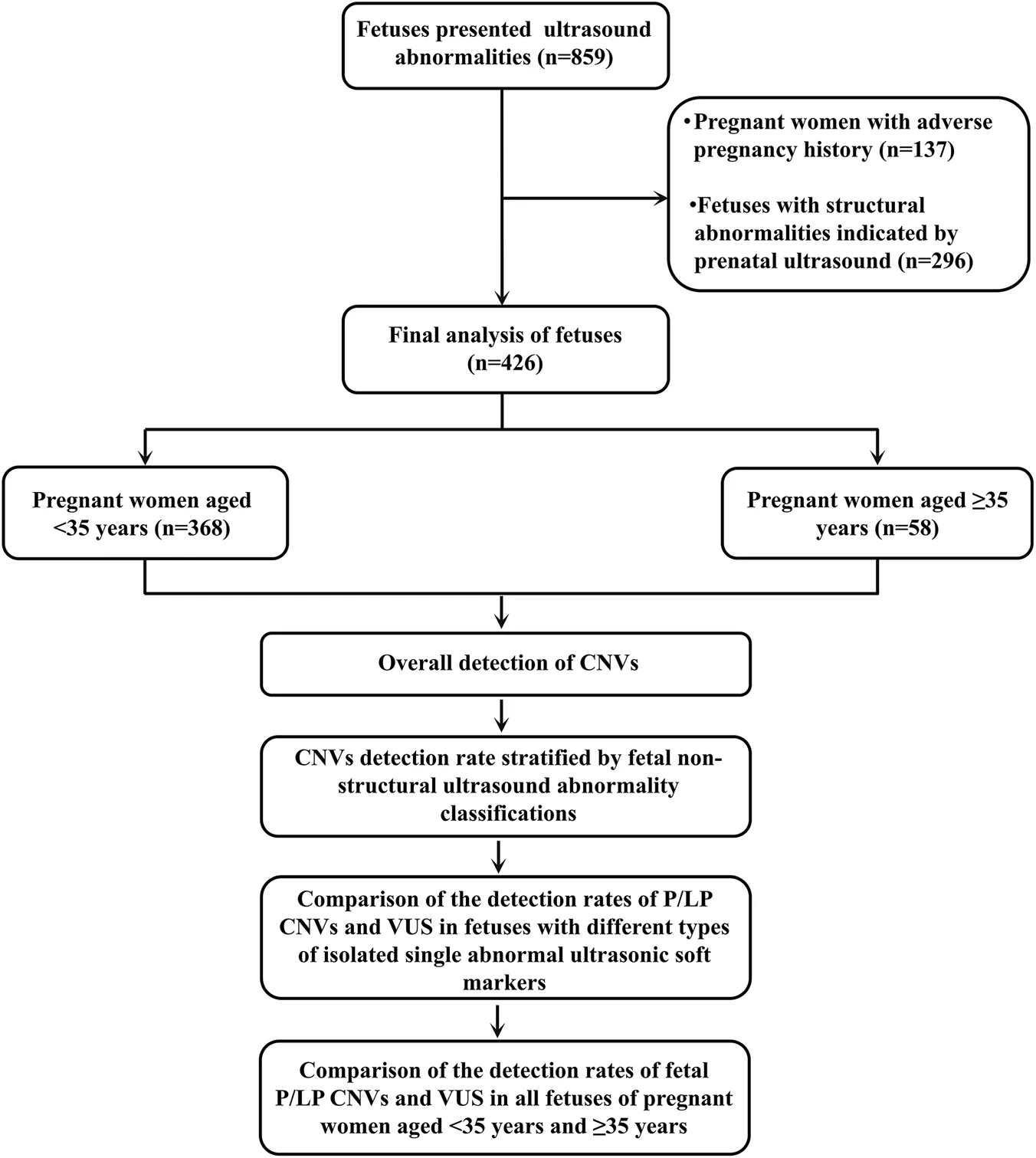
The flowchart of this study.

### Classification of fetal non-structural ultrasound abnormalities

#### Abnormal ultrasound soft markers

Abnormal ultrasound soft markers included increased nuchal translucency (NT) thickness, ventriculomegaly, nasal bone hypoplasia, choroid plexus cyst, short long bones, renal pelvic dilatation, echogenic bowel, subependymal cysts, aberrant subclavian artery, single umbilical artery, tricuspid regurgitation, persistent left superior vena cava, persistent right umbilical vein, increased nuchal fold (NF) thickness, and echogenic intracardiac focus. Diagnostic criteria for fetal abnormal ultrasound soft markers (Pan et al., 2023; Hu et al., 2021):

1. Increased NT thickness: NT thickness ≥3.0 mm at gestational age of 11–13^+6^ weeks.
2. Ventriculomegaly: At any gestational week, the width of the atrium of lateral ventricle exceeds 10 mm.
3. Nasal bone hypoplasia: Absence of visualized ossified nasal bone or shortened nasal bone length.
4. Choroid plexus cyst: Anechoic cystic structures identified within the echogenic choroid plexus of lateral ventricles between 16 and 24 weeks of gestation.
5. Short long bones: Femur or humerus length below the 10th percentile for corresponding gestational age.
6. Renal pelvic dilatation: Anteroposterior diameter of renal pelvis measuring 0.6–1.0 cm.
7. Echogenic bowel: Bowel echoes with signal intensity comparable to or higher than echoes of adjacent fetal skeletal structures.
8. Subependymal cysts: Distinct avascular anechoic cystic lesions located under the ventricular ependyma, without progressive ventricular dilatation.
9. Aberrant right subclavian artery: The right subclavian artery arises as the terminal branch of the proximal descending aorta and travels posterior to the trachea, after the origin of left common carotid artery and left subclavian artery from the aortic arch.
10. Single umbilical artery: Only one umbilical artery is visualized beside the fetal urinary bladder.
11. Tricuspid regurgitation: Doppler echocardiography in the apical four-chamber view reveals systolic backward blood flow crossing the tricuspid valve from the right ventricle to the right atrium.
12. Persistent left superior vena cava: Failure of regression of the left common cardinal vein, leading to the formation of persistent left superior vena cava.
13. Persistent right umbilical vein: The right umbilical vein remains patent accompanied by obliteration of the left umbilical vein.
14. Increased nuchal fold (NF): Nuchal fold thickness ≥6 mm in fetuses after 15 weeks of gestation.
15. Echogenic intracardiac focus: Isolated punctate echogenic foci within the ventricular cavity, exhibiting echo intensity equivalent to fetal osseous tissue.

#### Amniotic fluid abnormalities

Abnormal amniotic fluid volume is categorized as polyhydramnios when the deepest vertical pocket (DVP) ≥8 cm and/or amniotic fluid index (AFI) ≥24 cm, whereas oligohydramnios is diagnosed with DVP ≤2 cm and/or AFI <8 cm (Shi et al., 2023b; Simonyi et al., 2021).

#### Fetal growth restriction

Fetal growth restriction is defined as an estimated fetal weight below the 10th percentile for corresponding gestational age via ultrasonography, with or without abnormal umbilical or middle cerebral artery Doppler parameters (Rottenstreich et al., 2026).

Fetal ultrasound non-structural abnormalities collectively encompass abnormal ultrasonic soft markers, fetal growth restriction, and amniotic fluid volume anomalies (Luo et al., 2021; Huang et al., 2022). All enrolled fetuses were categorized based on prenatal ultrasound findings into four groups: abnormal ultrasound soft markers, fetal growth restriction, abnormal amniotic fluid volume, and cases with two or more combined non-structural ultrasound abnormalities.

### Sample collection and processing, CNV detection and classification

Invasive prenatal sampling was performed by experienced clinicians under ultrasound guidance. Chorionic villus sampling (CVS) was performed between 10 and 12 weeks of gestation. Amniotic fluid specimens (10 mL) were obtained from 15 to 22 gestational weeks, while umbilical cord blood samples (1.0–2.0 mL) were collected at 18–28 weeks of gestation. All specimens were immediately transported to the molecular genetics laboratory under standard refrigerated conditions. Genomic DNA was extracted using commercial DNA extraction kits following the manufacturer’s standard protocols. The concentration and purity of extracted DNA were detected by ultraviolet spectrophotometer. DNA samples with qualified concentration and purity were used for subsequent detection. All samples were tested via quantitative fluorescence polymerase chain reaction (QF-PCR) to rule out maternal cell contamination.

CMA served as the detection method for fetal CNVs. Chorionic villus samples, amniotic fluid cells and umbilical cord blood were utilized for genomic DNA extraction. Restriction enzyme NspI was employed to shear the genomic DNA into short fragments. Following amplification and biotin labeling, the DNA materials were thoroughly blended with hybridization solution and denatured. Next, the samples were incubated on Affymetrix Cytoscan 750k arrays to finish hybridization, washing and staining steps. Scanned raw microarray data were analyzed and interpreted systematically using GenomeStudio. All findings were cross-checked with globally acknowledged genomic variation repositories. The pathogenic potential of candidate CNVs was assessed with support from authoritative platforms: Clinical Genome Resource (ClinGen), Database of Genomic Variation and Phenotype in Humans using Ensembl Resources (DECIPHER), Database of Genomic Variants (DGV), Online Mendelian Inheritance Database in Man (OMIM), and the PubMed literature database. Based on the professional guidelines from ACMG, these copy number variants were classified into five clinical grades: pathogenic (P), likely pathogenic (LP), variants of uncertain significance (VUS), likely benign (LB), and benign (B) (Riggs et al., 2020; Brandt et al., 2020).

### Statistical analysis

SPSS was applied for all statistical processing in the present research. Enumeration data were described with case numbers and proportional values. We used the χ^2^ test or Fisher’s exact test to assess differences in the distribution of Pathogenic/likely pathogenic (P/LP) CNVs and VUS, covering comparisons of various single ultrasound soft anomalies and the two groups divided by age of the pregnant women. Statistical significance was set at *p* < 0.05.

## Results

### Baseline characteristics of study population

A total of 426 pregnant women and their fetuses were enrolled in this study. Among all participants, 368 (86.4%) pregnant women were younger than 35 years old, while 58 (13.6%) were aged 35 years or older. In terms of gestational age at sample collection, 56 cases (13.1%) were sampled at ≤13 weeks of gestation, 293 cases (68.8%) at 14–28 weeks, and 77 cases (18.1%) after 28 weeks of gestation. Regarding fetal specimen types, amniotic fluid samples accounted for the largest proportion with 370 cases (86.9%), followed by villus samples (55 cases, 12.9%), and only 1 case (0.2%) was umbilical cord blood sample (Table 1).

**TABLE 1:** General characteristics of pregnant women and the fetuses.

Characteristics	Cases (n = 426)
Age of pregnant women (years)	​
<35, n (%)	368 (86.4%)
≥35, n (%)	58 (13.6%)
The gestational age at the time of sampling (weeks)	​
≤13, n (%)	56 (13.1%)
14–28, n (%)	293 (68.8%)
>28, n (%)	77 (18.1%)
Types of fetuses samples	​
Villus, n (%)	55 (12.9%)
Amniotic fluid, n (%)	370 (86.9%)
Umbilical cord blood	1 (0.2%)

### Distribution of fetal non-structural ultrasound abnormalities

All enrolled fetuses presented with non-structural ultrasound abnormalities, which were classified into four categories (Figure 2). Abnormal ultrasound soft markers were the most common manifestation, occurring in 354 fetuses (83.1%). Fetal growth restriction was detected in 52 fetuses (12.2%). Abnormal amniotic fluid volume was found in 18 fetuses (4.2%), and two or more combined non-structural ultrasound abnormalities were observed in merely two fetuses (0.5%).

**FIGURE 2:**
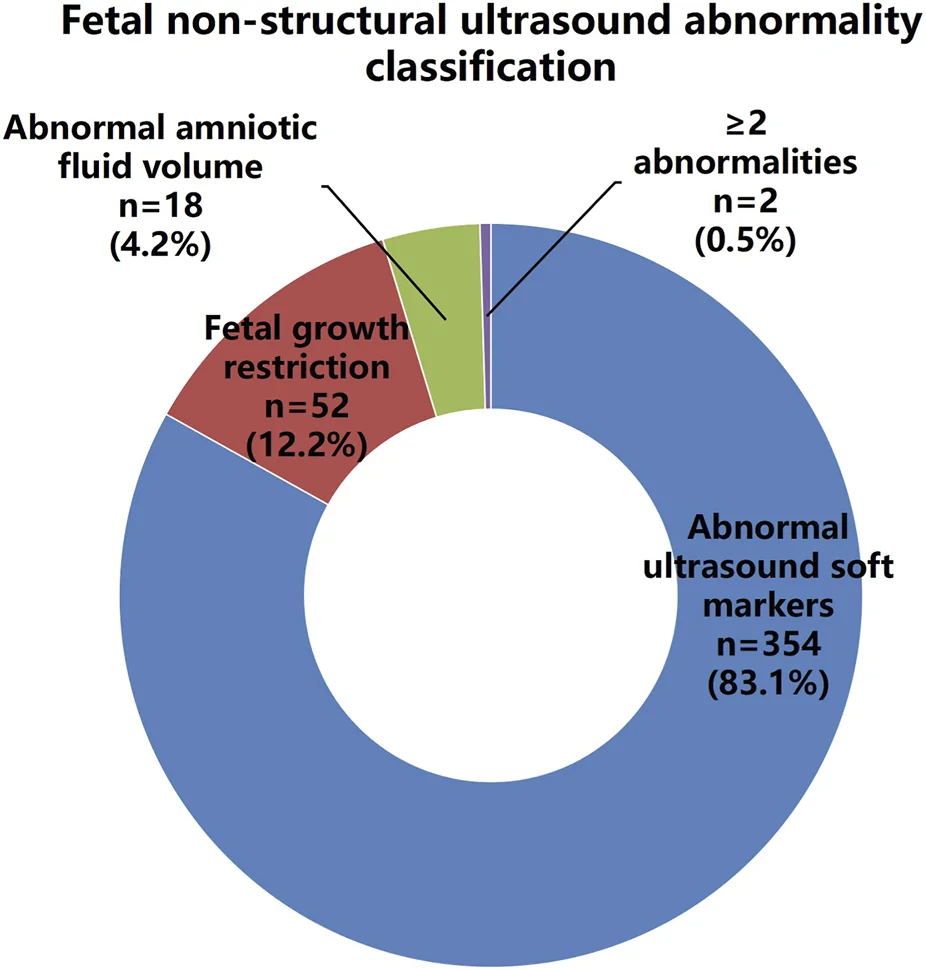
Fetal non-structural ultrasound abnormality classification.

### Overall detection results of CNVs

CNV analysis was performed for all 426 fetal samples. P/LP CNVs were identified in 19 fetuses (4.5%), and VUS were detected in 39 fetuses (9.2%). No P/LP CNVs or VUS were found in the remaining 368 fetuses, accounting for 86.4% of the total cases (Figure 3).

**FIGURE 3:**
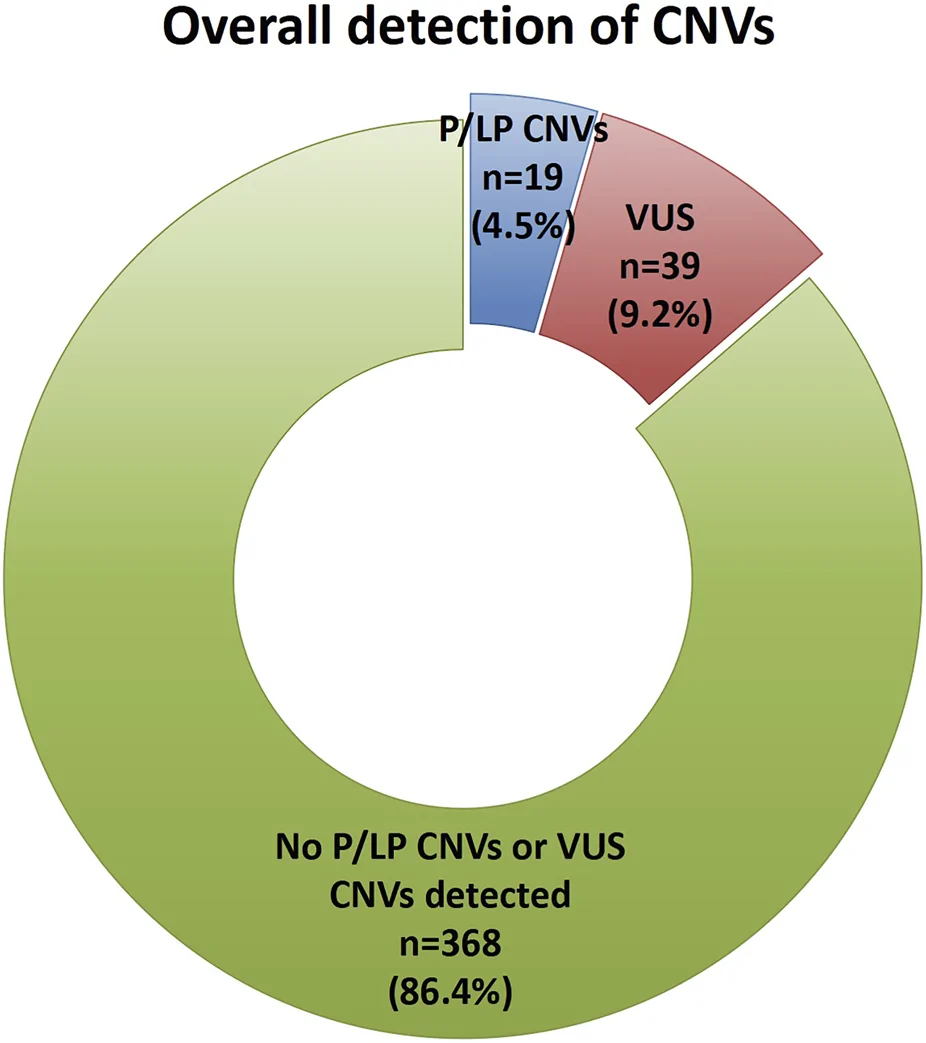
Overall detection of CNVs. Note: CNV, copy number variant; P/LP CNV, Pathogenic/Likely pathogenic CNV; VUS, variants of uncertain significance.

### CNV detection rates stratified by types of non-structural ultrasound abnormalities

The detection rates of P/LP CNVs and VUS were further analyzed across different categories of fetal non-structural ultrasound abnormalities (Figure 4). For fetuses with abnormal ultrasound soft markers, the detection rates of P/LP CNVs and VUS were 3.4% and 9.6%, respectively. In cases of fetal growth restriction, the positive rates of P/LP CNVs and VUS both reached 9.6%. Among fetuses with abnormal amniotic fluid volume, the P/LP CNVs detection rate was 5.6%, and no VUS were detected.

**FIGURE 4:**
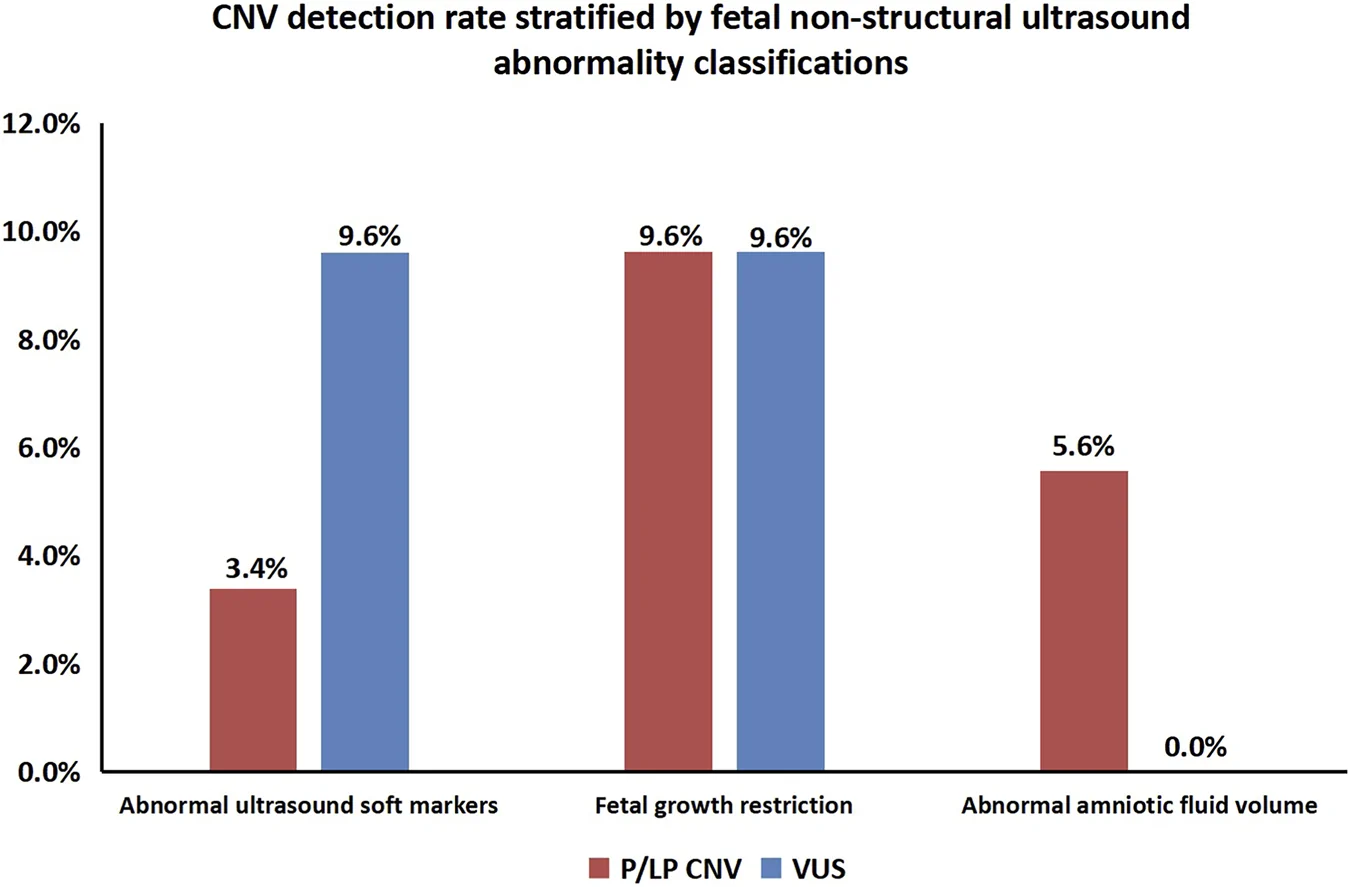
CNVs detection rate stratified by fetal non-structural ultrasound abnormality classifications. Note: CNV, copy number variant; P/LP CNV, Pathogenic/Likely pathogenic CNV; VUS, variants of uncertain significance.

### CNV detection rates of different abnormal ultrasound soft markers

Subgroup analysis on various single abnormal ultrasound soft markers revealed distinct CNVs positive rates (Figure 5). Persistent right umbilical vein, persistent left superior vena cava, and renal pelvic dilatation showed the highest CNVs detection rate at 33.3%. The detection rate was 25.0% for bilateral subependymal cysts, 17.9% for short long bones, 14.3% for increased NT thickness, 12.3% for ventriculomegaly, 12.2% for nasal bone hypoplasia, and 8.3% for choroid plexus cyst. By contrast, echogenic bowel, single umbilical artery, aberrant subclavian artery, tricuspid regurgitation, echogenic intracardiac focus, and increased nuchal fold thickness had a CNVs detection rate of 0%. Notably, some soft marker abnormalities with small sample sizes were excluded, such as persistent right umbilical vein (n = 3), persistent left superior vena cava (n = 3), and subependymal cysts (n = 4) (the blue bars in Figure 5), the CNVs detection rate was highest in fetuses with renal pelvic dilatation (33.3%, 5/15), followed by those with short long bones (17.9%, 5/28) and increased NT thickness (14.3%, 17/119).

**FIGURE 5:**
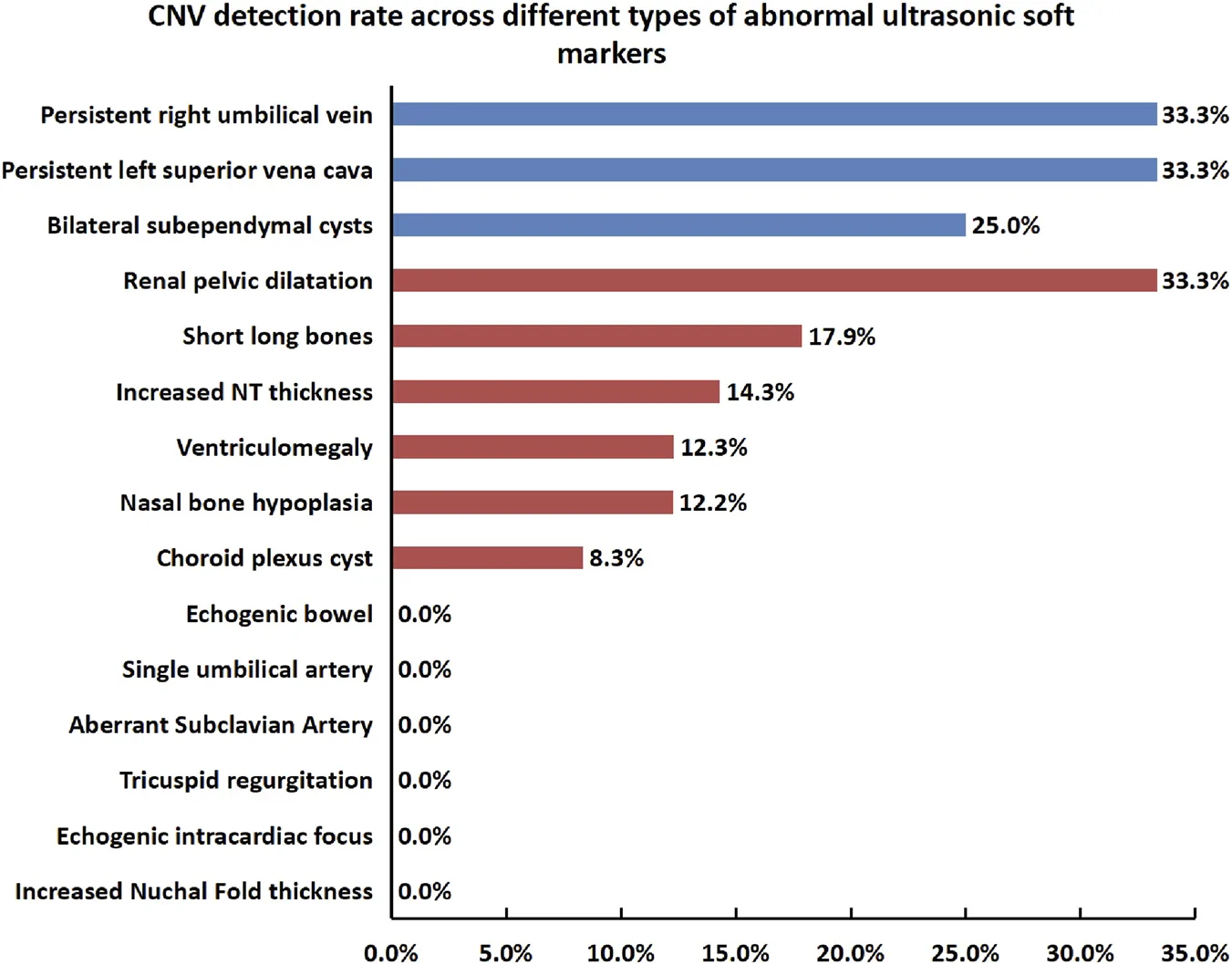
CNVs detection rate across different types of abnormal ultrasound soft markers. Note: NT, nuchal translucency; Blue bar chart: The types of ultrasound soft markers abnormalities in fetuses with P/LP CNVs or VUS and <5 cases with these ultrasound soft markers; Red bar chart: The types of ultrasound soft indicators abnormalities in fetuses with P/LP CNVs or VUS and ≥5 cases with these ultrasound soft markers.

### Comparison of CNVs detection rates among different groups

#### Correlation between maternal age and CNVs detection in fetuses with single abnormal ultrasound soft markers

We compared the combined detection rates of P/LP CNVs and VUS in fetuses with single abnormal ultrasound soft markers between pregnant women aged <35 years (n = 277) and those aged ≥35 years (n = 46) (Table 2). For each type of single soft marker abnormality, no statistically significant difference in the combined positive rate of P/LP CNVs and VUS was observed between the two maternal age groups (all *p* > 0.05). For indicators with limited cases in the advanced maternal age group such as echogenic bowel, bilateral subependymal cysts, aberrant subclavian artery, increased nuchal fold thickness and echogenic intracardiac focus, statistical comparison was not performed due to insufficient sample size.

**TABLE 2:** Comparison of the detection rates of P/LP CNVs and VUS in fetuses of pregnant women aged <35 years and ≥35 years with different types of isolated single abnormal ultrasonic soft markers.

Single ultrasonic soft marker abnormalities	Number of cases		P/LP CNVs and VUS		
	Pregnant women aged <35 years (n = 277)	Pregnant women aged ≥35 years (n = 46)	Pregnant women aged <35 years	Pregnant women aged ≥35 years	*p* values
Increased NT thickness	102	17	15 (14.7%)	2 (11.8%)	0.748
Ventriculomegaly	54	3	7 (13.0%)	0 (0)	0.506
Nasal bone hypoplasia	43	6	5 (11.6%)	1 (16.7%)	0.724
Choroid plexus cyst	20	4	2 (10.0%)	0 (0)	0.509
Short long bones	22	6	5 (22.7%)	0 (0)	0.198
Renal pelvic dilatation	13	2	4 (30.8%)	1 (50.0%)	0.591
Echogenic bowel	9	0	0 (0)	—	—
Bilateral subependymal cysts	4	0	1 (25.0%)	—	—
Aberrant subclavian artery	3	0	0 (0)	—	—
Single umbilical artery	3	2	0 (0)	0 (0)	—
Tricuspid regurgitation	0	2	—	0 (0)	—
Persistent left superior vena cava	2	1	1 (50.0%)	0 (0)	0.386
Persistent right umbilical vein	2	1	1 (50.0%)	0 (0)	0.386
Increased nuchal fold thickness	0	1	—	0 (0)	—
Echogenic intracardiac focus	0	1	—	0 (0)	—

CNV, copy number variant; P/LP CNV, Pathogenic/Likely pathogenic CNV; VUS, variants of uncertain significance.

#### Comparison of fetal CNVs status between different maternal age groups

The overall detection rates of P/LP CNVs and VUS were compared between pregnant women aged <35 years (n = 368) and ≥35 years (n = 58) (Table 3). P/LP CNVs were detected in 19 fetuses (5.2%) from younger pregnant women, whereas no P/LP CNVs were found in fetuses of women aged ≥35 years, with no significant inter-group difference (*p* = 0.090). The detection rate of VUS was 9.5% in the younger maternal group and 6.9% in the advanced maternal age group, and the difference was also not statistically significant (*p* = 0.631). Of note, these observations are restricted to this pre-selected cohort of fetuses with non-structural ultrasound abnormalities and cannot be extrapolated to the general obstetric population. The small sample size of the ≥35-year subgroup and zero detected P/LP CNVs in this group also limit the statistical power of this comparison.

**TABLE 3:** Comparison of the detection rates of fetal P/LP CNVs and VUS in fetuses of pregnant women aged <35 years and ≥35 years, respectively.

Groups	Pregnant women aged <35 years (n = 368)	Pregnant women aged ≥35 years (n = 58)	*p* values
P/LP CNVs	19 (5.2%)	0 (0)	0.090
VUS	35 (9.5%)	4 (6.9%)	0.631

CNV, copy number variant; P/LP CNV, Pathogenic/Likely pathogenic CNV; VUS, variants of uncertain significance.

Detailed clinical data of the 58 fetuses carrying P/LP CNVs or VUS, including maternal age, sampling gestational age, ultrasound findings, CMA results, variant clinical significance, and protein coding genes, are presented in Table 4. Notably, several genomic regions harbored recurrent CNVs across multiple fetuses in this cohort. The most frequent recurrent CNV was the 15q11.2 microdeletion, detected in five unrelated fetuses. Recurrent Xp22.31 deletions were observed in four fetuses, and 16p11.2-related pathogenic CNVs were identified in two fetuses. Other recurrent variants included 22q11.2-region CNVs detected in two fetuses. Different fetal ultrasound phenotypes were observed among fetuses carrying these identical-locus CNVs. Parental testing was unavailable for the majority of cases in the present study.

**TABLE 4:** The clinical data of 58 fetuses with P/LP or VUS CNVs were detected by CMA analysis.

Number	Age of pregnant woman (years)	The gestational age at the time of sampling (weeks)	Types of ultrasound abnormalities	CMA results	Clinical significance of CNVs	Protein coding genes
1	33	13^+2^	Increased NT thickness, single umbilical artery	arr [GRCh37]10q26.11q26.3 (121223476–135426386) × 1 (14.20 Mb)	P	89 genes
2	32	31	Fetal growth restriction	arr [GRCh37]3q11.2q12.1 (95527472–98345123) × 3 (2.82 Mb)	VUS	*EPHA6, ARL6, CRYBG3, RIOX2, GABRR3, OR5A,OR5H, OR5K, CLDND1, GPR15, CPOX*
3	26	31^+5^	Ventriculomegaly	arr [GRCh37]16p12.2 (21405328–21816543) × 1 (411 Kb)	VUS	*NPIPB3, METTL9, IGSF6, OTOA*
4	23	28^+6^	Ventriculomegaly	arr [GRCh37]Xp22.31 (6449837–8143509) × 1 dn (1.69 Mb)	P	*VCX3A, PUDP, STS, VCX, PNPLA4, VCX2*
5	25	12^+5^	Increased NT thickness	arr [GRCh37]22q11.21q11.23 (21804597–23654007) × 3 (1.85 Mb)	VUS	70 genes
6	31	16^+3^	Increased NT thickness	arr [GRCh37]4q12q13.1 (58193592–62730657) × 3 (4.54 Mb)	VUS	*ADGRL3*
7	26	26^+4^	Fetal growth restriction	arr [GRCh37]22q11.21 (18631365–21800471) × 1 (3.17 Mb)	P	54 genes
8	26	21^+5^	Renal pelvic dilatation	arr [GRCh37]Xp21.1 (31713418–31825390) × 0 (112 Kb)	P	*DMD*
9	27	13	Increased NT thickness	arr [GRCh37]3q13.31q22.1 (116764010–129606827) × 2 hmz (12.84 Mb)	P	110 genes
10	33	18	Nasal bone hypoplasia	arr [GRCh37]15q15.3 (43868571–44007537) × 1 (139 kb)	VUS	*CATSPER2, CKMT1A, CKMT1B, PPIP5K1, STRC*
11	33	12	Increased NT thickness	arr [GRCh37]5q14.2 (81492684–82000597) × 3 (508 kb)	VUS	*ATG10, RPS23*
12	30	28	Ventriculomegaly	arr [GRCh37]2q13 (110498142–110980295) × 3 (482 kb)	VUS	*LIMS3, MALL, MTLN, NPHP1, RGPD5*
13	27	30^+4^	Fetal growth restriction	arr [GRCh37]4q35.2 (187929332–188943890) × 3 (1.02 Mb)	VUS	*ZFP42*
14	29	26^+4^	Short long bones	arr [GRCh37] 6q27 (169848646–170270972) × 3 (422 kb)	VUS	*WDR27, C6orf120, PHF10, DYNLT2, ERMARD*
15	26	12	Increased NT thickness	arr [GRCh37] 5q35.3 (178730385–178936592) × 3 (206 kb)	VUS	*ADAMTS2*
16	26	30	Fetal growth restriction	arr [GRCh37] 1p36.33 (1413423–2059598) × 3 (646 kb) arr [GRCh37] 2q21.1 (130926994–131194508) × 3 (268 kb)	VUS	*ATAD3B, ATAD3A, TMEM240, SSU72, FNDC10, MIB2, MMP23B, CDK11B, SLC35E2B, CDK11A, NADK, GNB1, CALML6, TMEM52, CFAP74, GABRD, PRKCZ;* *SMPD4, MZT2B, TUBA3E, VMA22, IMP4, PTPN18*
17	36	23	Renal pelvic dilatation	arr [GRCh37] 5p12p11 (45647745–46389261) × 3 (742 kb)	VUS	*HCN1*
18	24	24	Subependymal cysts	arr [GRCh37] Xq28 (148565633–148884123) × 3 (318 kb)	VUS	*IDS, EOLA1, HSFX3, MAGEA9B, TMEM185A, MAGEA11, MAGEA9*
19	27	19	Renal pelvic dilatation	arr [GRCh37]22q11.22 (22311349–22579064) × 1 (268 kb)	VUS	*TOP3B*
20	32	20^+5^	Increased NT thickness	arr [GRCh37]15q11.2 (22770422–23625785) × 3 (855 kb); arr [GRCh37]Xq28 (151872967–152087536) × 3 (215 kb)	VUS	*TUBGCP5, CYFIP1, NIPA2, NIPA1, GOLGA8S;* *MAGEA2B, MAGEA12, CSAG1, MAGEA2, MAGEA6, CETN2, NSDHL, ZNF185*
21	29	12^+5^	Increased NT thickness	arr [GRCh37]15q15.3 (43868571–44007537) × 1 (139 kb)	VUS	*CATSPER2, CKMT1A, CKMT1B, PPIP5K1, STRC*
22	27	12^+6^	Increased NT thickness	arr [GRCh37]2q24.1q31.1 (159577115–177258204) × 2 hmz (17.68 Mb); arr [GRCh37]16p13.3p12.3 (94808–17409998) × 2 hmz (17.32 Mb)	VUS	96 genes 250 genes
23	26	12^+6^	Increased NT thickness	arr [GRCh37]16p11.2 (29428532–30190029) × 1 (761 kb)	P	*BOLA2-SMG1P6, BOLA2, SLX1B, SLX1B-SULT1A4, SULT1A4, NPIPB12, SPN, QPRT, C16orf54, ZG16, KIF22, MAZ, PRRT2, PAGR1, MVP, CDIPT, SEZ6L2, ASPHD1, KCTD13, TMEM219, TAOK2, HIRIP3, INO80E, DOC2A*
24	25	18^+2^	Ventriculomegaly	arr [GRCh37]Xp22.31 (6,455,152–8135568) × 1 (1.68 Mb)	P	*PUDP, STS, VCX, PNPLA4*
25	38	17^+1^	Increased NT thickness	arr [GRCh37]16p13.3p13.2 (6896958–9417288) × 3 (2.52 Mb)	VUS	*RBFOX1, TMEM114, METTL22, ABAT, TMEM186, PMM2, CARHSP1, USP7, HAPSTR1*
26	29	12^+2^	Increased NT thickness	arr [GRCh37]16p13.11 (15338153–16327887) × 3 (990 Kb)	P	*NPIPA5, MPV17L, BMERB1, MARF1, NDE1, MYH11, CEP20, ABCC1, ABCC6, NOMO3*
27	28	32^+2^	Fetal growth restriction	arr [GRCh37]15q11.2 (22770422–23288350) × 1 (518 kb)	P	*TUBGCP5, CYFIP1, NIPA2, NIPA1*
28	28	24^+6^	Nasal bone hypoplasia	arr [GRCh37]12q21.31 (82919621–86264470) × 3 (3.34 Mb)	VUS	*TMTC2, SLC6A15, TSPAN19, LRRIQ1, ALX1, RASSF9*
29	29	18^+1^	Increased NT thickness, fetal growth restriction	arr [GRCh37]Xp22.31 (6,455,152–8135568) × 1 (1.68 Mb)	P	*PUDP, STS, VCX, PNPLA4*
30	27	17	Increased NT thickness	arr [GRCh37]Xp22.33 (1358802–3520663) × 3 (2.16 Mb)	VUS	*CSF2RA, IL3RA, SLC25A6, ASMTL-AS1, ASMTL, P2RY8, AKAP17A, ASMT, DHRSX, ZBED1, CD99, XG, GYG2, ARSD, ARSD-AS1, ARSL, ARSH, ARSF, MXRA5, SNORA48B*
31	27	19^+6^	Nasal bone hypoplasia	arr [GRCh37]15q11.2 (22770422–23082237) × 1 (312 Kb)	P	*TUBGCP5, CYFIP1, NIPA2, NIPA1*
32	32	29^+1^	Short long bones	arr [GRCh37]10q26.3 (134639293–135426386) × 1 (787 Kb)	VUS	*ADGRA1-AS1, ADGRA1, KNDC1, UTF1, VENTX, ADAM8, TUBGCP2, ZNF511, CALY, PRAP1, FUOM, ECHS1, PAOX, MTG1, SPRN, CYP2E1, SYCE1*
33	26	27^+4^	Ventriculomegaly	arr [GRCh37]Yp11.2 (6135281–9171486) × 0 (3.04 Mb)	VUS	*AMELY, TBL1Y*
34	27	27	Fetal growth restriction	arr [GRCh37]1p36.33p11.2 (888659–121339317) × 2 hmz (120.45 Mb); arr [GRCh37]1q21.2q44 (149879545–249198164) × 2 hmz (99.32 Mb)	VUS	1050 genes 897 genes
35	27	25^+3^	Polyhydramnios	arr [GRCh37]15q11.2 (22770422–23214655) × 1 (444 Kb)	P	*TUBGCP5, CYFIP1, NIPA2, NIPA1*
36	27	31^+1^	Renal pelvic dilatation	arr [GRCh37]22q11.23 (23652587–25041592) × 3 (1.39 Mb)	VUS	*FAM230I, PCAT14, IGLL1, DRICH1, RGL4, ZNF70, VPREB3, C22orf15, CHCHD10, MMP11, SMARCB1, DERL3, SLC2A11, MIF-AS1, MIF, GSTT2B, DDTL, DDT, GSTT2, GSTT4, GSTT1-AS1, GSTT1, CABIN1*
37	27	12^+2^	Increased NT thickness	arr [GRCh37]12p11.1 (33430047–34599314) × 3 (1.17 Mb)	VUS	*SYT10, ALG10*
38	28	16^+5^	Increased NT thickness	arr [GRCh37]1q44 (248003188–248771545) × 1 (768 Kb)	VUS	*TRIM58, OR2*
39	31	35^+6^	Fetal growth restriction	arr [GRCh37]15q11.2q13.1 (23290788–28540345) × 1 (5.25 Mb)	P	*GOLGA8S, GOLGA6L2, MKRN3, MAGEL2, NDN, PWRN4, PWRN2, PWRN3, PWRN1, NPAP1, SNHG14, SNRPN, SNURF, SNORD107, PWARSN, PWAR5, SNORD64, SNORD108, PWAR6, SNORD109A, SNORD116*
40	32	25^+2^	Persistent left superior vena cava	arr [GRCh37]1q21.1 (145406788–145888926) × 3 (482 Kb)	VUS	*HJV, TXNIP, POLR3GL, ANKRD34A, LIX1L, LIX1L-AS1, RBM8A, PEX11B, ITGA10, ANKRD35, PIAS3, NUDT17, POLR3C, RNF115, CD160, PDZK1, GPHRA*
41	28	31^+3^	Short long bones	arr [GRCh37]2q13 (111371702–113111856) × 1 (1.74 Mb)	LP	*BUB1, SNORD132, ACOXL, ACOXL-AS1, BCL2L11, ANAPC1, MERTK, TMEM87B, FBLN7, ZC3H8, ZC3H6*
42	30	30^+4^	Fetal growth restriction	arr [GRCh37]4q35.2 (187933402–188943890) × 3 (1.01 Mb); arr [GRCh37]5q23.1 (116670621–118148274) × 3 (1.48 Mb)	VUS	*ZFP42*; No RefSeq gene
43	26	29	Ventriculomegaly	arr [GRCh37]16p11.2 (28708187–29090030) × 1 (382 Kb)	P	*NPIPB9, ATXN2L, TUFM, SH2B1, ATP2A1, ATP2A1-AS1, RABEP2, CD19, NFATC2IP, SPNS1, LAT*
44	32	13^+3^	Increased NT thickness	arr [GRCh37]1p36.11 (24360737–24867196) × 1 (506 Kb); arr [GRCh37]4q12q13.1 (58221562–62730657) × 3 (4.51 Mb)	VUS	*MYOM3, IL22RA1, IFNLR1, GRHL3, STPG1, NIPAL3, RCAN3AS;* *ADGRL3*
45	33	24^+2^	Nasal bone hypoplasia	arr [GRCh37]15q21.3q26.3 (56282641–102429040) × 2∼3 months (46.15 Mb)	P	323 genes
46	28	17^+4^	Increased NT thickness	arr [GRCh37]15q11.2 (22770422–23082237) × 1 (312 Kb)	P	*TUBGCP5, CYFIP1, NIPA2, NIPA1*
47	33	17^+1^	Choroid plexus cyst	arr [GRCh37]19q12 (29568991–30605808) × 1 (1.04 Mb)	VUS	*UQCRFS1, VSTM2B, POP4, PLEKHF1, C19orf12, CCNE1, URI1*
48	28	25	Short long bones	arr [GRCh37]4q23 (99562179–100431609) × 1 (869 Kb)	VUS	*TSPAN5, EIF4E, METAP1, ADH5, ADH4, ADH6, ADH1A, ADH1B, ADH1C, ADH7*
49	35	16^+2^	Increased NT thickness	arr [GRCh37]Xq13.1 (68025398–68961225) × 2 (936 Kb)	VUS	*EFNB1, PJA1, NALF2, EDA*
50	22	27^+6^	Short long bones	arr [GRCh37]16p13.3p11.2 (94808–31935367) × 2 hmz (31.84 Mb)	VUS	449 genes
51	34	32^+4^	Fetal growth restriction	arr [GRCh37]15q11.2 (22770422–23282798) × 1 (512 Kb)	P	*TUBGCP5, CYFIP1, NIPA2, NIPA1*
52	26	23^+1^	Persistent right umbilical vein	arr [GRCh37]18q12.1 (26834461–28442180) × 3 (1.61 Mb)	VUS	No RefSeq gene
53	24	25^+6^	Ventriculomegaly	arr [GRCh37]8p23.3 (1125565–2193914) × 3 (1.07 Mb)	VUS	*DLGAP2-AS1, CLN8, ARHGEF10, KBTBD11-OT1, KBTBD11, MYOM2*
54	29	17^+1^	Choroid plexus cyst	arr [GRCh37]19q12q13.33 (30841000–51279882) × 2 hmz (20.44 Mb)	VUS	524 genes
55	36	22	Nasal bone hypoplasia	arr [GRCh37]12q21.31 (83844900–86021386) × 3 (2.18 Mb); arr [GRCh37]Xp22.31p22.2 (9166253–10190141) × 3 (1.02 Mb)	VUS	*SLC6A15, TSPAN19, LRRIQ1, ALX1;* *TBL1X, GPR143, SHROOM2, CLDN34, WWC3, CLCN4*
56	29	22^+2^	Nasal bone hypoplasia	arr [GRCh37]7p21.3p15.2 (8745176–26439331) × 2 hmz (17.69 Mb); arr [GRCh37]20q11.21q13.12 (29510307–45556516) × 2 hmz (16.05 Mb)	VUS	61 genes; 211 genes
57	29	18^+3^	Renal pelvic dilatation	arr [GRCh37]21q21.1 (22372647–22982857) × 1 (610 Kb)	VUS	*NCAM2*
58	28	32^+2^	Fetal growth restriction	arr [GRCh37]Xp22.31 (6578896–7918931) × 0 (1.34 Mb)	P	*PUDP, STS, VCX, PNPLA4*

CMA, chromosome microarray analysis; CNV, copy number variant; P CNV, Pathogenic CNV; LP CNV, Likely pathogenic CNV; VUS, variants of uncertain significance.

## Discussion

Prenatal ultrasound serves as the most fundamental and widely applied screening tool throughout gestation, enabling real-time evaluation of fetal development and early recognition of atypical fetal phenotypes (Sriraam et al., 2024; Vepa et al., 2024). Non-structural ultrasound abnormalities refer to subtle imaging manifestations that do not constitute definitive fetal organ malformations but are closely correlated with fetal chromosomal aberrations and genomic disorders (Huang et al., 2022; Lan et al., 2024). In routine clinical practice, these findings frequently prompt clinicians to recommend invasive prenatal genetic testing (Cherian et al., 2022; Emms et al., 2022). Notably, the majority of existing relevant studies have focused on mixed populations receiving prenatal diagnosis, including women with recurrent spontaneous abortion, stillbirth, adverse neonatal outcomes, familial genetic disorders or multiple risk factors (Huang et al., 2024; Wang et al., 2026; Wang C. et al., 2022). However, clinical evidence regarding fetal CNVs among low-risk pregnant women without any adverse pregnancy history remains insufficient and inconsistent. In the present single-center study with prospective participant recruitment and retrospective data analysis, we enrolled 426 pregnancies in pregnant women with no adverse pregnancy history complicated by fetal non-structural ultrasound abnormalities, systematically analyzed the distribution characteristics of P/LP CNVs and VUS, and further explored the associations between CNV detection results, different ultrasonic phenotypes and maternal age. The findings supplement clinical data for prenatal genetic risk assessment, stratified management and standardized genetic counseling in pregnant women with no adverse pregnancy history.

The demographic distribution of the enrolled participants reflected the current clinical characteristics of prenatal diagnosis practice. Most women receiving genetic testing for fetal non-structural ultrasound anomalies were of childbearing age <35 years. Specimen collection was largely concentrated in the second trimester, which corresponds to the optimal time window for routine prenatal ultrasound screening and invasive genetic testing in clinical settings (Sterpu et al., 2019). Amniocentesis was the predominant sampling approach in this cohort, whereas chorionic villus sampling and cord blood collection were applied far less frequently, consistent with the clinical application scope and safety profile of different invasive prenatal techniques. This distribution is highly consistent with the routine workflow of prenatal diagnosis in domestic and international clinical centers (Zhang et al., 2022; Kayhan et al., 2025). Amniocentesis performed in the second trimester is the most common invasive sampling method due to its stable operation, low complication rate and wide application scope. Meanwhile, the dominance of women under 35 years old reflects the current demographic characteristics of childbearing women and indicates that non-structural ultrasound abnormalities are not confined to advanced-age pregnancies, but are also prevalent in young low-risk gravidas (Yan et al., 2024; Nordklev et al., 2025).

Among all enrolled fetal ultrasonic phenotypes, abnormal ultrasound soft markers were the predominant manifestation, accounting for 83.1% of total cases, whereas fetal growth restriction, abnormal amniotic fluid volume and combined multiple non-structural abnormalities occupied 12.2%, 4.2% and 0.5% respectively. This distribution is in accordance with previous epidemiological observations of prenatal ultrasound abnormalities (Chen et al., 2024; Zhou et al., 2022). As the most commonly detected non-structural anomalies in prenatal screening, various ultrasound soft markers are the primary reason for clinicians to initiate further genetic testing in pregnancies (Mangla and Kumar, 2025). The overall detection rate of P/LP CNVs was 4.5%, and VUS was detected in 9.2% of fetuses; up to 86.4% of cases presented no clinically significant CNVs. Compared with studies targeting high-risk pregnant populations, the positive rate of clinically meaningful CNVs in this low-risk cohort was markedly lower (Lan et al., 2024; Chen et al., 2024). High-risk groups with adverse pregnancy history inherently carry elevated genetic susceptibility, which inevitably increases the detection rate of pathogenic genomic variations. In contrast, for women with uncomplicated obstetric history, isolated fetal non-structural ultrasound abnormalities represent a relatively moderate genetic risk. This result well explains the ongoing debate on whether to implement universal CNVs screening for low-risk pregnancies with single non-structural ultrasound anomalies. On one hand, these ultrasound signs cannot be completely ignored due to the definite risk of pathogenic CNVs; on the other hand, universal invasive genetic testing will bring unnecessary economic burden and psychological stress to most low-risk families. Therefore, establishing a stratified risk assessment system is particularly critical for this population.

Stratified analysis by categories of non-structural ultrasound abnormalities revealed prominent heterogeneity in CNVs detection rates. Fetuses with fetal growth restriction achieved the highest detection rates of P/LP CNVs and VUS (both 9.6%). Fetal growth restriction is a complex adverse fetal phenotype affected by placental function, maternal physical status and fetal genetic background (Salmeri et al., 2022). Accumulating evidence has confirmed that unexplained fetal growth restriction is strongly correlated with hidden genomic abnormalities, even in low-risk pregnancies (Shi et al., 2022; Nowakowska et al., 2021). Our data further verify that fetal growth restriction, compared with other non-structural anomalies, is a stronger warning indicator for pathogenic CNVs, which should be fully emphasized in prenatal counseling. For fetuses with abnormal ultrasound soft markers, the detection rates of P/LP CNVs and VUS were 3.4% and 9.6%, respectively. In the subgroup with abnormal amniotic fluid volume, the P/LP CNVs detection rate was 5.6%, no VUS was identified. Abnormal amniotic fluid volume is more frequently related to placental dysfunction, fetal kidney and urinary tract temporary dysplasia (Schulz et al., 2023). This difference in etiological composition leads to its low risk of pathogenic CNVs. Collectively, different types of non-structural ultrasound abnormalities carry distinct genetic risks, and one-size-fits-all testing strategies are not applicable. Clinicians need to combine specific ultrasonic findings to conduct graded risk notification and formulate individualized testing recommendations.

When subdivided into single types of ultrasound soft markers, the CNVs detection rates showed dramatic differences across different indicators. The data of this study revealed that among subtypes with sufficient sample sizes presenting abnormal ultrasonic soft markers, fetuses with renal pelvic dilatation had the highest detection rate of CNVs at 33.3%, followed by short long bones (17.9%) and increased NT thickness (14.3%). In contrast, the detection rates of CNVs were relatively low for ventriculomegaly and nasal bone dysplasia, at 12.3% and 8.3% respectively. Previous clinical studies have confirmed that increased NT thickness is a well-recognized ultrasonic soft marker strongly associated with fetal chromosomal abnormalities and genomic variations, and the degree of NT elevation is positively correlated with the risk of CNVs (Vo et al., 2024; Yang et al., 2024). The steady positive detection rate in the increased NT thickness group in this study further supports this finding, indicating that this marker is of great reference value for screening occult fetal genomic abnormalities. Short long bones and renal pelvic dilatation are common ultrasonic soft anomalies in fetuses, which were previously mostly regarded as benign physiological variations (Akalın et al., 2023; Viaux-Savelon et al., 2012). However, the relatively high CNVs detection rates observed in this study suggest that isolated soft marker abnormalities of these types are not entirely free of genetic risks, as they are still accompanied by a certain probability of genomic copy number variations, which warrants close clinical attention.

Maternal age is a classic independent risk factor for fetal chromosomal and genomic diseases. Advanced maternal age (≥35 years) has long been proven to increase the risk of fetal aneuploidy and *de novo* genomic variations (Picchetta et al., 2025). Nevertheless, the correlation between maternal age and CNVs detection in low-risk pregnancies with fetal non-structural ultrasound abnormalities has not been fully clarified. In our study, the overall P/LP CNVs detection rate was 5.2% in women younger than 35 years, while no P/LP CNVs were detected in the advanced maternal age group; the VUS rates were 9.5% and 6.9% in the two groups respectively. Statistical analysis demonstrated no significant inter-group differences for either P/LP CNVs or VUS (*p* > 0.05). Further grouping analysis based on single ultrasound soft markers also showed that the combined positive rates of P/LP CNVs and VUS did not differ significantly between the two age subgroups. We speculate that the strict inclusion of women without adverse pregnancy history is the core reason for this result. This low-risk population excludes confounding factors such as recurrent miscarriage, family genetic history and repeated fetal anomalies, which are closely linked to high genetic risk. Within this relatively homogeneous low-risk cohort, advanced maternal age fails to further elevate the risk of fetal CNVs in fetuses with non-structural ultrasound abnormalities. This conclusion complements the existing cognition of maternal age and fetal genomic disorders, and suggests that for fetuses with non-structural ultrasound anomalies in pregnant women with no adverse pregnancy history, maternal age should not be regarded as a decisive factor for CNVs testing. It should be highlighted that our maternal-age-related results are limited to this selected cohort with non-structural ultrasound abnormalities and are not directly applicable to unselected general pregnancies, given the relatively small sample size of the advanced-maternal-age subgroup.

VUS is an inevitable part of CNVs detection in prenatal diagnosis. Due to the limitations of current genomic databases and research progress, the pathogenicity of VUS cannot be clearly defined, which has become a major challenge in prenatal genetic counseling (Wafik et al., 2024). In our cohort, the overall detection rate of VUS reached 9.2%. Although these variants have not been confirmed to be pathogenic, they may bring long-term psychological burden to pregnant families. Combined with our subgroup data, VUS was widely distributed in various non-structural ultrasound abnormalities, especially in cases with fetal growth restriction and abnormal soft markers. Therefore, when reporting CNVs results, genetic counselors need to fully interpret the uncertainty of VUS, avoid excessive alarm, and guide families to choose follow-up strategies rationally.

Several limitations of this study need to be acknowledged objectively. First, this is a single-center study, and the enrolled population is limited to patients from a single medical institution. Potential selection bias cannot be completely ruled out, and the extrapolation of the conclusions needs to be verified by large-sample, multi-center prospective studies. Second, the sample size of some rare ultrasound soft markers and the advanced maternal age subgroup was relatively small. A few subgroups failed to perform effective statistical comparison, which may affect the comprehensiveness of subgroup analysis. Third, this study only focused on prenatal ultrasound phenotypes and CNVs detection results, and lacked long-term postnatal follow-up of fetal growth, development and neurological function. Consequently, the long-term clinical significance of detected VUS and mild CNVs cannot be further evaluated. Fourth, Limited availability of parental CMA segregation testing also restricted our ability to re-evaluate the clinical significance of several VUS cases. In addition, all ultrasound findings were extracted from routine clinical reports without centralized prospective image review, which may still limit full inter-study comparability.

## Conclusion

In summary, for pregnant women without adverse pregnancy history, fetal non-structural ultrasound abnormalities present distinct genetic risks across different phenotypes. Fetal growth restriction and several ultrasound soft markers are correlated with higher CNVs detection rates, while most common soft markers have a low risk of pathogenic genomic variations. Within this low-risk population, maternal age does not significantly affect the detection rates of fetal P/LP CNVs and VUS. Our research results support the implementation of stratified risk management and individualized genetic counseling for low-risk pregnancies with fetal non-structural ultrasound abnormalities. On the premise of ensuring prenatal diagnosis safety, it helps to reduce excessive medical testing, optimize the allocation of medical resources, and provide practical reference for the formulation of prenatal diagnosis guidelines for low-risk populations.

## Data Availability

The original contributions presented in the study are included in the article/supplementary material, further inquiries can be directed to the corresponding authors.
